# GeOKG: geometry-aware knowledge graph embedding for Gene Ontology and genes

**DOI:** 10.1093/bioinformatics/btaf160

**Published:** 2025-04-11

**Authors:** Chang-Uk Jeong, Jaesik Kim, Dokyoon Kim, Kyung-Ah Sohn

**Affiliations:** Department of Software and Computer Engineering, Ajou University, Suwon, 16499, South Korea; Department of Biostatistics, Epidemiology, and Informatics, Perelman School of Medicine, University of Pennsylvania, Philadelphia, PA 19104, USA; Institute for Biomedical Informatics, University of Pennsylvania, Philadelphia, PA 19104, USA; Institute for Biomedical Informatics, University of Pennsylvania, Philadelphia, PA 19104, USA; Department of Bioengineering, University of Pennsylvania, Philadelphia, PA 19104, USA; Department of Biostatistics, Epidemiology, and Informatics, Perelman School of Medicine, University of Pennsylvania, Philadelphia, PA 19104, USA; Institute for Biomedical Informatics, University of Pennsylvania, Philadelphia, PA 19104, USA; Department of Software and Computer Engineering, Ajou University, Suwon, 16499, South Korea; Department of Artificial Intelligence, Ajou University, Suwon, 16499, South Korea

## Abstract

**Motivation:**

Leveraging deep learning for the representation learning of Gene Ontology (GO) and Gene Ontology Annotation (GOA) holds significant promise for enhancing downstream biological tasks such as protein–protein interaction prediction. Prior approaches have predominantly used text- and graph-based methods, embedding GO and GOA in a single geometric space (e.g. Euclidean or hyperbolic). However, since the GO graph exhibits a complex and nonmonotonic hierarchy, single-space embeddings are insufficient to fully capture its structural nuances.

**Results:**

In this study, we address this limitation by exploiting geometric interaction to better reflect the intricate hierarchical structure of GO. Our proposed method, Geometry-Aware Knowledge Graph Embeddings for GO and Genes (GeOKG), leverages interactions among various geometric representations during training, thereby modeling the complex hierarchy of GO more effectively. Experiments at the GO level demonstrate the benefits of incorporating these geometric interactions, while gene-level tests reveal that GeOKG outperforms existing methods in protein–protein interaction prediction. These findings highlight the potential of using geometric interaction for embedding heterogeneous biomedical networks.

**Availability and implementation:**

https://github.com/ukjung21/GeOKG.

## 1 Introduction

Gene Ontology (GO) is the most widely used ontology, offering a structured and computable knowledge base of gene functions across species (Ashburner *et al.* 2000). GO organizes information into three primary domains—biological processes (BP), cellular components (CC), and molecular functions (MF)—each represented as a Directed Acyclic Graph (DAG) where nodes denote GO terms and edges capture their interrelations. To model the complexity of biological phenomena, GO incorporates five distinct relation types—“is a,” “part of,” “regulates,” “positively regulates,” and “negatively regulates”—which enable nuanced representation of functional interdependencies.

In recent years, embedding techniques for ontologies have evolved along three main perspectives: graphical, logical, and lexical. Graph-based approaches, such as GOA2Vec (Zhong and Rajapakse 2020), Anc2Ve (Edera *et al.* 2022), and HiG2Vec (Kim *et al.* 2021), leverage the inherent structure of the GO graph to preserve relationships like ancestor–descendant links and subontology memberships. However, by treating the ontology as an unlabeled network, these methods often miss the deeper semantics embedded in formal axioms. To address this, logical embedding methods incorporate ontology axioms from description logic into the learning process. The EL Embeddings approach, for instance, constructs a geometric model that satisfies EL++ logic constraints, defining bespoke scoring functions to enforce that if an axiom A⊑B holds, then the embedding of A lies within that of B (Kulmanov *et al.* 2019, Jackermeier *et al.* 2024). Such model-theoretic embeddings treat the vector space as an interpretation of the ontology, representing each concept as a region, such as an n-dimensional ball or box, and ensuring that inclusion relations between regions mirror the ontology’s hierarchy (Althagafi *et al.* 2024). Other methods take a lexical angle by leveraging textual information: Onto2Vec (Smaili *et al.* 2018) and OPA2Vec (Smaili *et al.* 2019), e.g. use corpora of ontology axioms augmented with entity names, definitions, and comments to learn embeddings, effectively combining formal structure with contextual word embeddings. Building on these ideas, (Chen *et al.* 2021) introduced OWL2Vec*, which encodes the semantics of an ontology by taking into account its graph structure, lexical information, and logical constructors.

Parallel to these ontology-specific strategies, a broad class of knowledge graph embedding (KGE) methods have been developed for general relational data. KGE models embed entities and relations into low-dimensional vector spaces by optimizing scoring functions over factual triples. Many extensions have been built by exploiting either more sophisticated spaces or more sophisticated operations. For instance, MuRE and MuRP introduce Euclidean versus Poincaré hyperbolic geometry for embeddings, with MuRP outperforming its Euclidean counterpart on benchmarks by better modeling hierarchy (Balazevic *et al.* 2019). Recent models also incorporate more complex relational operations (Supplementary Section S3). RotE/RotH apply rotational components in Euclidean versus hyperbolic space, RefE/RefH add reflections, and AttE/AttH integrate attention mechanisms for relation-specific operations (Chami *et al.* 2020). These advanced KGE approaches can capture certain hierarchical or logical patterns in data, yet they still treat an ontology with a single geometric space, which can be limiting when faced with the intrinsic structural diversity of ontological graphs.

**Figure 1.**
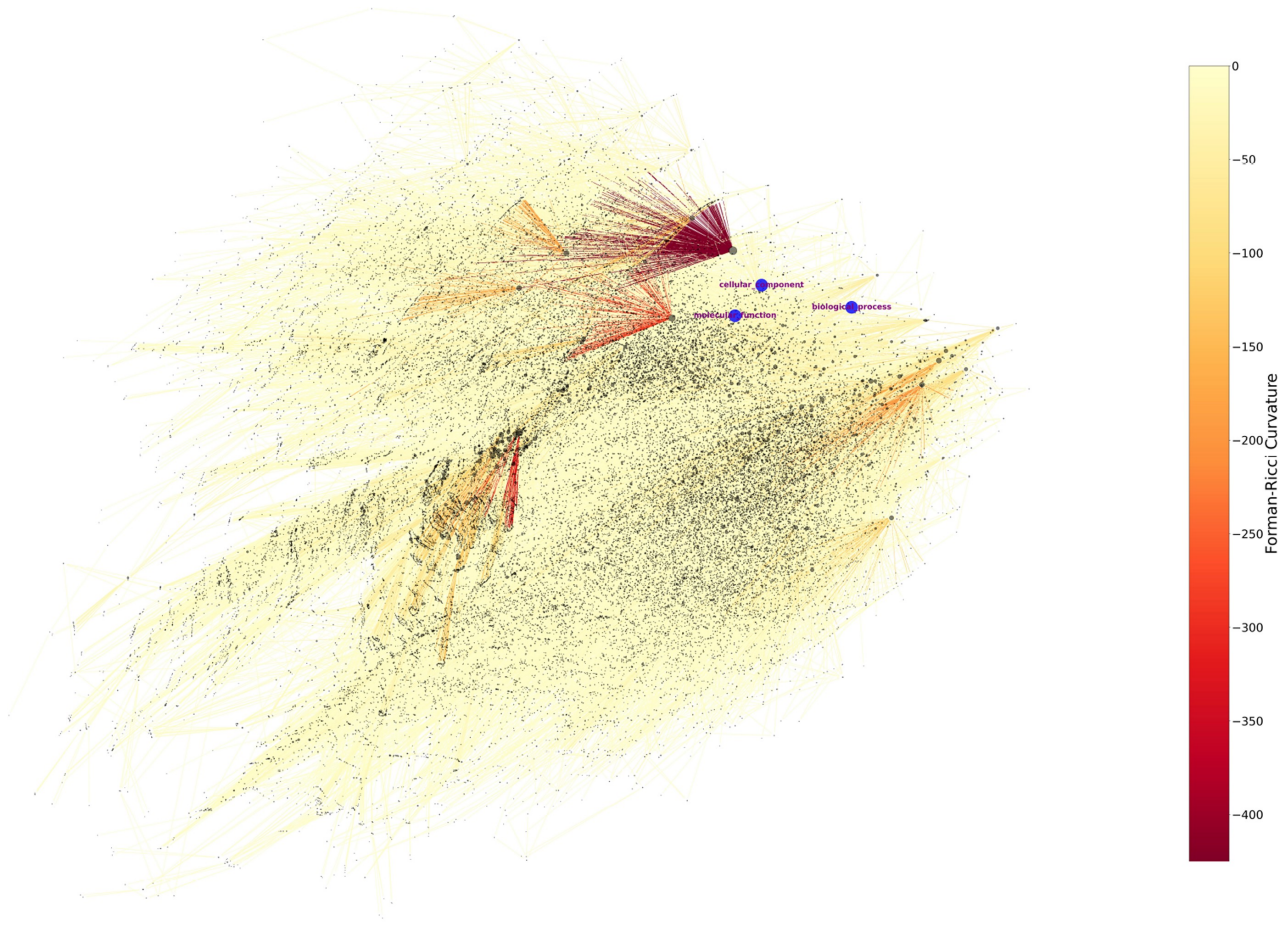
Forman-Ricci curvature of Gene Ontology graph. Everydot represents the node of the graph, with larger dots indicating more connections. Directed edges appear as arrows. This compact visualization underscores the graph’s structural and geometric nuances, where contains broad range of curvature. A detailed explanation of Forman-Ricci curvature is provided in Supplementary Section S1.

As shown in (Fig. 1), indeed, the GO graph exhibits heterogeneous curvature characteristics. The diverse range of curvature values observed in the GO graph not only underscores its complex, heterogeneous architecture but also has important implications for embedding strategies. The simultaneous presence of highly negative curvature alongside near-neutral curvature implies that the network exhibits a multifaceted connectivity pattern. Rather than relying on a single geometric space for embedding, these heterogeneous characteristics suggest that using multiple geometric spaces and exploring their interactions can yield more flexible and structurally faithful embeddings. Additionally, recent studies on real-world knowledge graphs have highlighted the benefits of using mixed-curvature spaces or incorporating interactions between different geometries for embedding heterogeneous and complex graph (Gu *et al.* 2018, Zhu *et al.* 2020, Cao *et al.* 2022).

**Figure 2.**
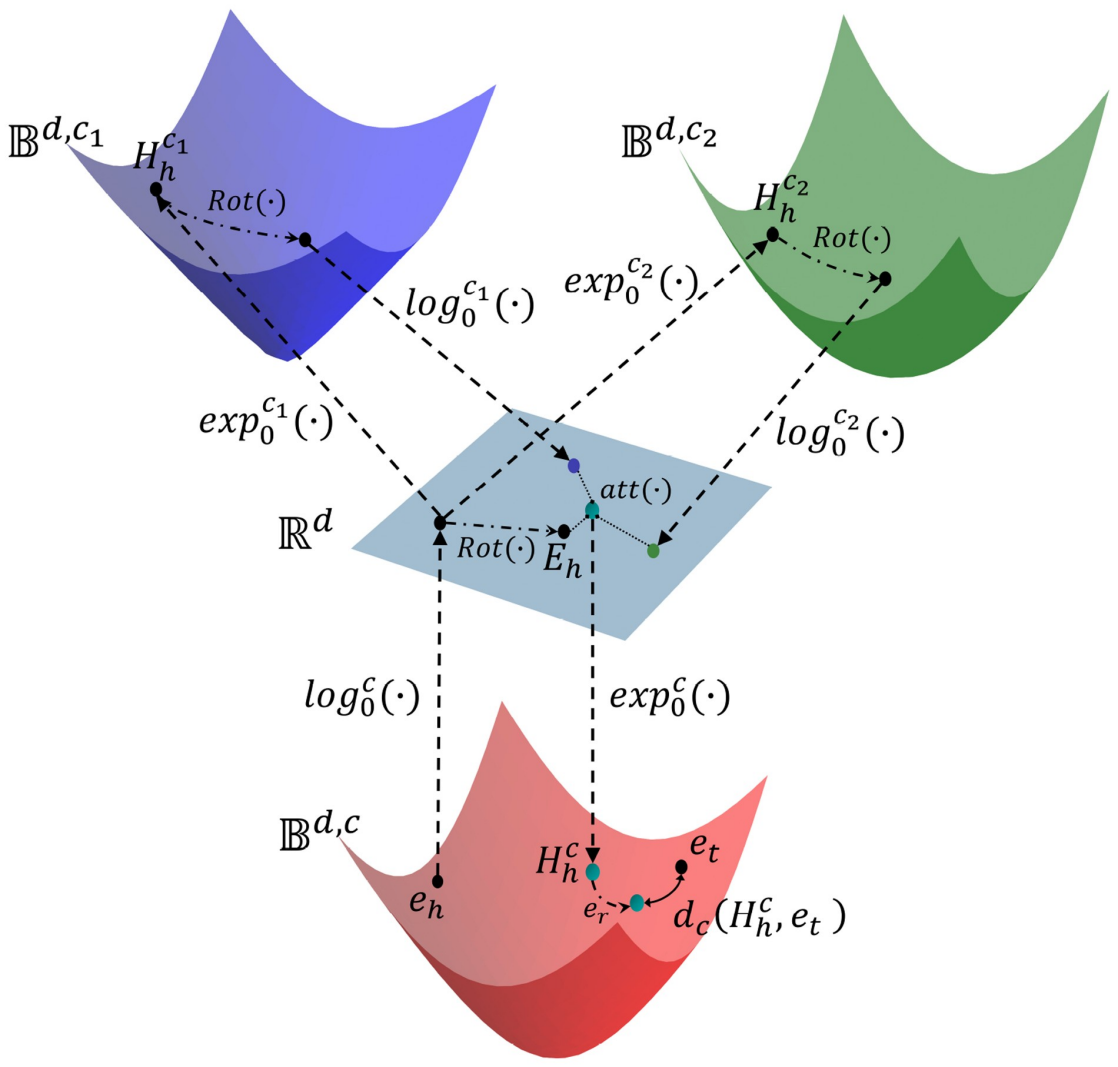
Schematic of GeOKG-H. GeOKG-H is the hyperbolic space embedding model for GO. $e_{h}$ and $e_{t}$ are the final embeddings of head and tail entities, respectively. The approach uses exponential and logarithmic mappings (Supplementary Section S2.1), exp(·) and log(·), to bridge between Euclidean and hyperbolic spaces. In each interaction space, the rotational transformation Rot(·) is applied and then the results are integrated back into the Euclidean space. The attention mechanism, att(·), aggregates the geometric information from all the interaction spaces. The learning objective in hyperbolic space with learnable curvature *c* is to optimize the entity and relation embeddings by minimizing the hyperbolic distance $d_{c}(H_{h}^{c},e_{t})$.

Motivated by these insights, we propose Geometry-aware knowledge graph embedding for Gene Ontology and genes (GeOKG), a novel method that captures graph geometry by utilizing information from various topological spaces to learn vector representations for GO terms and genes. This approach uses a knowledge graph embedding (KGE) framework, which maps entities and relations into low-dimensional vectors while preserving their semantic meanings. We especially utilize the KGE method that integrates Euclidean and hyperbolic geometries, harnessing the concept of geometry interaction (Fig. 2). By learning relational embeddings across multiple topological spaces, the model capture richer relational semantics compared to learning in a single geometric space. Additionally, GeOKG can be flexibly extended to various graphs by adapting the embedding space or altering the combination of interaction spaces for geometry interaction, according to the graph’s structural characteristics.

Our approach enhances the ability to capture the intricacies of GO and gene semantics through the geometry interaction mechanism. Extensive evaluations on both GO-level and gene-level tasks show that our method outperforms state-of-the-art methods. Furthermore, visualizations demonstrate that our embeddings can generalize to new, unseen data. The codes and trained gene embeddings are available at https://github.com/ukjung21/GeOKG.

## 2 Background and preliminaries

### 2.1 Knowledge graph embeddings

Knowledge Graph Embedding (KGE) translates complex, multi-relational data in knowledge graphs into a low-dimensional vector space. In KGE, head-relation-tail triples (h,r,t)∈E⊆V×R×V are used, where V and R represent the sets of entities and relations, respectively. U denotes a topological space that is used in KGE. The goal is to represent entities v∈V by vectors $e_{v}\in U^{d}$ and relations r∈R by vectors $e_{r}\in U^{d}$ in a chosen embedding space, so that $e_{h}*e_{r}\approx e_{t}$ if the triple (h,r,t) is true, where ∗ is a relational operation. Enhancements in the field of KGE have been achieved by using more sophisticated topological spaces aligned with the graph’s intrinsic structure, from Euclidean to hyperbolic space (Balazevic *et al.* 2019, Chami *et al.* 2020).

### 2.2 Hyperbolic geometry

Non-Euclidean geometry diverges from the classical Euclidean view by allowing the parallel postulate to be false. Hyperbolic geometry is a form of non-Euclidean geometry with a negative curvature. This geometry is often modeled through the Poincaré ball model, which is a stereographic projection of hyperbolic space and provides a compact representation of hyperbolic geometry with negative curvature.

A point x in a d-dimensional hyperbolic space with curvature −c (c>0) is represented in the Poincaré ball model as $B^{d,c}=\{x\in R^{d}:||x|{|}^{2}<\frac{1}{c}\}$. The model also defines the tangent space at a point x, $T_{x}B^{d,c}$, which contains all possible directions of the path in the manifold leaving from x. In this work, the connection between the hyperbolic space and its tangent space is established through the exponential and logarithmic maps at the origin, defined as:

(1) $${\;\text{exp}\;}_{0}^{c}(v)=\text{tanh}(\sqrt{c}||v||)\frac{v}{\sqrt{c}||v||},$$

 

(2) $${\;\text{log}\;}_{0}^{c}(y)=\text{arctanh}(\sqrt{c}||y||)\frac{y}{\sqrt{c}||y||}.$$

where $v\in T_{0}B^{d,c}$, and ||·|| denotes the Euclidean norm. The distance between two points in hyperbolic space is defined along a geodesic, which is the shortest path between those points. The distance formula in the Poincaré ball model is given by:

(3) dc(x,y)=2ctanh−1(c‖−x ⊕c y‖).

where ${⊕}_{c}$ represents Möbius addition, an addition in hyperbolic geometry (Supplementary Section S2.2).

## 3 Materials and methods

### 3.1 GeOKG

Geometry interaction is essential for capturing the reliable structure of space through geometric message propagation, which engages different spatial properties of Euclidean and non-Euclidean spaces. In Cao *et al.* (2022), which proposed a geometric interaction KGE model, three geometric spaces are typically used: Euclidean, hyperbolic, and hyperspherical. However, since GO DAGs do not contain cyclic structures, GeOKG uses one Euclidean space and two distinct hyperbolic spaces with different curvatures ($-c_{1}$ and $-c_{2}$) to enable interactions across the hyperbolic spaces with varying curvatures. The reason for this combination of interaction spaces is explained in detail in Supplementary Section S5.5. These spaces, defined by their respective spatial metrics, propagate geometric information using exponential and logarithmic mappings, enabling interaction across different spaces.

The methodology of GeOKG-H can be summarized into four main steps (Fig. 2):

1. Initialize the head and tail embeddings($e_{h}$, $e_{t}$) in a hyperbolic space with curvature −c. And then use the logarithmic map to project the head entity to Euclidean space.
2. Use exponential maps to project the head entity from Euclidean space $R^{d}$ to hyperbolic interaction spaces $B^{d,c_{1}}$ and $B^{d,c_{2}}$.
3. In each space, apply rotational transformation Rot(·) and aggregate the results back into Euclidean space.
4. Use an attention mechanism, att(·) to fuse the geometric information from both Euclidean and hyperbolic spaces. And then project the result into the hyperbolic space with curvature −c ($H_{h}^{c}$).
5. In the hyperbolic space with curvature −c, apply the translational operation with $e_{r}$ to $H_{h}^{c}$ so that it moves closer to the tail entity $e_{t}$.

For a head entity h, the embedding transformations in the interaction spaces are given by:

-
$E_{h}=\mathrm{Rot}(\Theta_{r}){\;\text{log}\;}_{0}^{c}(e_{h})$ for the Euclidean space embedding,
-
$H_{h}^{c_{1}}=\mathrm{Rot}(\Theta_{r}){\;\text{exp}\;}_{0}^{c_{1}}({\;\text{log}\;}_{0}^{c}(e_{h}))$ for the first hyperbolic space embedding, where ${\;\text{exp}\;}_{0}^{c_{1}}({\;\text{log}\;}_{0}^{c}(e_{h}))$ (with $c_{1}>0$) obtains the projection of the head entity into the first hyperbolic interaction space,
-
$H_{h}^{c_{2}}=\mathrm{Rot}(\Theta_{r}){\;\text{exp}\;}_{0}^{c_{2}}({\;\text{log}\;}_{0}^{c}(e_{h}))$ for the second hyperbolic space embedding, where ${\;\text{exp}\;}_{0}^{c_{2}}({\;\text{log}\;}_{0}^{c}(e_{h}))$ (with $c_{2}>0$) obtains the projection of the head entity into the second hyperbolic interaction space,

where Rot(·) and $\Theta_{r}$ are the rotational function and parameter, respectively (Supplementary Section S3). The interaction function is then defined as:

(4) $$\text{Inter}(E_{h},H_{h}^{c_{1}},H_{h}^{c_{2}})=\lambda_{E}E_{h}+\lambda_{H}^{1}{\;\text{log}\;}_{0}^{c_{1}}(H_{h}^{c_{1}})+\lambda_{H}^{2}{\;\text{log}\;}_{0}^{c_{2}}(H_{h}^{c_{2}}),$$

where λ is an attention vector and $\left(\lambda_{E},\lambda_{H}^{1},\lambda_{H}^{2}\right)=\text{Softmax}\left(\lambda^{T}E_{h},\lambda^{T}H_{h}^{1},\lambda^{T}H_{h}^{2}\right)$. Here, ${\;\text{log}\;}_{0}^{c_{1}}(\cdot )$ and ${\;\text{log}\;}_{0}^{c_{2}}(\cdot )$ map $H_{h}^{c_{1}}$ and $H_{h}^{c_{2}}$ into the tangent space, respectively, for geometric interaction. The scoring function, designed to assess the likelihood of triples for this geometric interaction, is defined as:

(5) ϕ(h,r,t)=−(dc(exp0c(Inter(Eh,Hhc1,Hhc2)) ⊕c er,et)+dc((exp0c(Inter(Et,Ht1,Ht2)) ⊕c er−1,eh))+b.

Here, $d_{c}(\cdot ,\cdot )$ denotes the Poincaré distance, b is a bias acting as a margin, and $e_{r}$  $e_{r^{-1}}$ are the embeddings for relation type r and its inverse, respectively.

For training, we use a cross-entropy loss minimization strategy with uniform negative sampling. Specifically, negative instances for a given triplet (h,r,t) are uniformly selected from all potential triplets formed by altering the tail entity. The loss function is given by:

(6) $$L=\sum_{\{h,r,t\}\in \Omega \cup \Omega^{\prime }}\;\text{log}\;\left(1+\text{exp}\;\left(-Y\phi (h,r,t)\right)\right),$$

where Ω represents the set of observed triplets and $\Omega^{\prime }=E\times R\times E-\Omega$ denotes the set of unobserved triplets. Here, Y∈{−1,1} corresponds to the label of the triplet (h,r,t), indicating its presence within Ω or otherwise. The explanation and schematic of GeOKG-E method is in Supplementary Section S5.1 and Supplementary Fig. S5. The hyperparameter settings for both GeOKG-H and GeOKG-E are described in Supplementary Section S5.2.

### 3.2 GO term embeddings

Let $V_{GO}$ and $R_{GO}$ be the sets of GO terms and relations, respectively. We extracted the GO term relationships from the GO dataset, processing them into triplets of the form (h,r,t), where $h,t\in V_{GO}$ and $r\in R_{GO}$. This dataset encompasses 83 975 interactions among 42 950 GO terms across 5 types of relationships (Supplementary Section S4.1). For GO-level task evaluation, we trained GO embeddings in Poincaré ball, $B^{d,c}=\{x\in R^{d}:||x|{|}^{2}<\frac{1}{c}\}$, and this approach is denoted as GeOKG-H. In GeOKG-E, we pre-trained GO embeddings in Euclidean space. The optimal embeddings of GO terms are learned by minimizing the cross-entropy loss with negative sampling (Equation 6).

(7) $$L_{GO}=\sum_{\{h,r,t\}\in \Omega_{GO}}(\mathrm{logsig}(\phi (h,r,t))+\sum_{t^{\prime }\in V_{GO}}lo\mathrm{gsig}(-\phi (h,r,t^{\prime }))),$$

where *logsig* is a logsigmoid function, $\text{log}\;\left(\frac{1}{1+e^{-x}}\right)$. $\Omega_{GO}$ denotes the set of GO triplets and $\left(h,r,t^{\prime }\right)$ denotes the uniformly sampled negative triplet by perturbing the tail entity. We randomly sampled 50 negative triplets per positive triplet.

### 3.3 Gene embeddings with pre-trained GO term embeddings

To learn gene embeddings, we first constructed a GOA dataset specifically for Homo sapiens that captures the associations between Gene Ontology (GO) terms and genes. The processed corpus comprises 18 137 genes and 286 628 interactions (Supplementary Section S4.2). Next, we derived optimal gene embeddings in Euclidean space by fine-tuning the pre-trained GO term embeddings. We applied the same loss function as used for the GO term embeddings, but with the dataset replaced by the GOA graph—composed of GO and GOA triplets. In this step, 50 negative examples were uniformly sampled from the positive triples, following the same strategy as before. This model is denoted as GeOKG-E, in contrast to GeOKG-H.

## 4 Results

### 4.1 GO level experiments: link prediction, link reconstruction and relation type prediction

The dataset is split into *train*($\Omega_{\text{train}}$), *valid*($\Omega_{\text{valid}}$) and *test* ($\Omega_{\text{test}}$) subsets. The dataset composition for GO-level experiments is described in more detail in Supplementary Section S4.1. The embeddings are generated by optimizing the scoring function ϕ:V×R×V→R [Equation (5)], which evaluates the plausibility of triples. We conducted a controlled comparison among models that utilize rotational relation operations (i.e. RotE, RotH, and GeOKG). This approach minimizes the confounding effects from different relational operations and demonstrates the impact of geometric interactions. A broader comparison with additional KGE models is detailed in Supplementary Section S6.1. For each method, we determined the optimal embedding dimension for every task through a grid search over d∈{100,200,300,500,1000}. We then selected the dimension that achieved the highest MRR in link prediction, the best F1 score in link reconstruction, and the highest Micro F1 in relation type prediction. U is the utilized space for vector embedding or geometry interaction. $R^{d}$ represents the Euclidean space and $B^{d}$ represents the Poincaré ball. The best results are in bold.

#### 4.1.1 Link prediction

The objective of link prediction is to enrich the KG by predicting true yet unobserved relationships using the scoring function in Equation 5. In this process, test triplets are evaluated by ranking them against all possible triplets generated by substituting masked entities with others from the knowledge graph, i.e. (h, r, ?). Consistent with existing KGE research (Bordes *et al.* 2013), we use two ranking-based metrics for evaluation: the mean reciprocal rank (MRR) and the hit rate at K (H@K). MRR averages the inverse rankings of correctly identified triplets, while H@K measures the ratio for which the correct triplet is present in the top K predictions. For GeOKG-H, geometry interaction involves both Euclidean space and Poincaré ball ($R^{d},B^{d}$), with the Poincaré ball being the final embedding space (Fig. 2). Other KGE models embed directly within a single geometric space, either Euclidean($R^{d}$) or hyperbolic($B^{d}$). Results show that GeOKG-H, which uses geometry interaction and hyperbolic space for embedding, outperforms other KGE methods that use a single geometry (Table 1).

**Table 1.** Link prediction results on Gene Ontology. The best results are in bold.

U	Model	Dim	MRR	H@1	H@10	H@50
$R^{d}$	RotE	1000	0.382	0.283	0.577	**0.706**
$B^{d}$	RotH	200	0.381	0.283	0.574	0.694
$R^{d},B^{d}$	**GeOKG-H**	300	**0.385**	**0.288**	**0.581**	0.704

#### 4.1.2 GO-level benchmark comparison

In addition to the standard GO-level link prediction experiment described in Section 4.1.1, we performed a supplementary evaluation aimed at directly comparing our method with existing GO embedding approaches. In this evaluation, the test set exclusively uses the “is_a” relation. The prediction task is defined as follows: given a head entity h and the fixed relation “is_a”—i.e. (h, “is_a,” ?)—the goal is to assign the highest score to the correct tail entity t. This experimental configuration replicates the evaluation protocols used in prior studies, thereby ensuring a fair comparison of embedding performance. Only models that can process triplets were included for comparison; graph-based models that treat edges as tuples were excluded. Our method outperformed all the previous ontology embedding methods (Table 2).

**Table 2.** Link prediction benchmark comparison. The best results are in bold.^a^

Model	Dim	MRR	H@1	H@10	H@50
Onto2Vec	200	0.025	0.007	0.058	0.202
OPA2Vec	200	0.078	0.027	0.176	0.446
EL Embeddings	100	0.027	0.003	0.066	0.203
Box2EL	200	0.036	0.001	0.101	0.290
OWL2Vec*	200	0.142	0.065	0.301	0.566
**GeOKG-H**	300	**0.242**	**0.074**	**0.449**	**0.605**

a For the previous models, we used the default dimensions reported in their respective publications.

#### 4.1.3 Link reconstruction

In link reconstruction experiments, the goal was to predict the existence of a link between two Gene Ontology (GO) terms. Negative pairs were generated by randomly sampling an equal number to the test set (Supplementary Section S4.1). For each pair—with h and t representing the head and tail GO terms, respectively—we computed a link score defined as $max_{r\in R_{GO}}(\phi (h,r,t))$, where $R_{GO}$ denotes the set of all GO relationship types and ϕ is the scoring function for a potential link. Notably, extending our link prediction experiments, the link reconstruction results demonstrated superior performance over all other KGE models across every evaluation metric (Table 3).

**Table 3.** Link reconstruction results on Gene Ontology. The best results are in bold.

U	Model	Dim	AUROC	AUPRC	F1 score
$R^{d}$	RotE	300	0.901	0.929	0.835
$B^{d}$	RotH	300	0.905	0.931	0.842
$R^{d},B^{d}$	**GeOKG-H**	1000	**0.945**	**0.956**	**0.868**

#### 4.1.4 Relation type prediction

In relation type prediction experiments, we aimed to predict the precise relation type existing between two Gene Ontology (GO) terms. The evaluation is conducted by verifying whether $r^{\prime }$, obtained from $r^{\prime }=\mathrm{argma}x_{r\in R_{GO}}\phi (h,r,t)$, matches r (Equation 5). To ensure a fair evaluation, instances where a single GO term pair was associated with multiple relation types were omitted from the test set. Furthermore, due to the imbalanced distribution of relation types within the GO, we report both macro and micro F1 scores to provide a comprehensive assessment of performance (Table 4). GeOKG-H, demonstrated superior performance among rotation-based knowledge graph embedding methods, indicating its effectiveness in identifying the most plausible relation type between two GO terms.

**Table 4.** Relation type prediction results on Gene Ontology. The best results are in bold.

U	Model	Dim	Macro F1	Micro F1
$R^{d}$	RotE	1000	0.846	0.920
$B^{d}$	RotH	300	0.851	0.906
$R^{d},B^{d}$	**GeOKG-H**	100	**0.865**	**0.924**

### 4.2 Protein–protein interaction prediction using gene embeddings

We leverage gene embeddings derived from the GOA corpus to perform protein–protein interaction (PPI) prediction. First, each gene embedding is mapped to its corresponding protein within the STRING PPI network, thereby creating protein embeddings that serve as the foundation for our prediction tasks. These protein embeddings are then fed into the prediction head, a two-layer multi-layer perceptron (MLP), that features a hidden layer with a ReLU nonlinear activation function. Notably, the final output layer of this MLP is adapted according to the specific prediction objective. Here, we then compare our approach against existing GO embedding techniques as well as the best-performing KGE methods in both Euclidean and hyperbolic spaces, highlighting the impact of the embedding spaces and geometric interactions. A broader comparison with additional KGE models is detailed in Supplementary Section S6.2.

#### 4.2.1 Binary interaction classification

In binary classification, we frame the detection of an interaction between two proteins as a binary classification task, training the model with Binary Cross-Entropy (BCE) loss. To ensure balance in the training data, we extract an equal number of positive and negative links from the STRING PPI dataset (Supplementary Section S4.3). For each protein pair, their respective embeddings are fed into the prediction head—described in detail earlier—which produces output logit values used to infer the presence or absence of an interaction. As shown in Table 5, our method, GeOKG-E, not only surpasses all previous GO embedding techniques but also outperforms other single-space-based KGE methods across all evaluation metrics.

**Table 5.** STRING binary interaction prediction. The best results are in bold.^a^

Model	Dim	AUROC	AUPRC	F1 score
Onto2Vec	200	0.9314	0.9313	0.8586
OPA2Vec	200	0.9629	0.9621	0.9051
EL-embeddings	100	0.9339	0.9336	0.8614
Box2EL	200	0.9724	0.9727	0.9189
OWL2Vec*	200	0.9633	0.9624	0.9033
GOA2Vec	150	0.9401	0.9402	0.8718
Anc2Vec	200	0.9712	0.9716	0.9194
HiG2Vec	1000	0.9787	0.9790	0.9301
RefE	1000	0.9836	0.9837	0.9414
RotH	1000	0.9830	0.9828	0.9398
GeOKG-E	1000	**0.9848**	**0.9849**	**0.9455**

a RefE and RotH are the best-performing KGE methods within the categories of Euclidean and hyperbolic embedding models, respectively.

#### 4.2.2 STRING physical interaction score prediction

Here, we predict the STRING-defined physical interaction score (ranging from 0 to 1000) as a regression problem using Mean Squared Error (MSE) loss. The proteins involved in physical connections are related within a protein complex (Szklarczyk *et al.* 2023). As shown in Table 6, GeOKG-E outperforms all other methods.

**Table 6.** STRING physical interaction score prediction. The best results are in bold.^a^

Model	Dim	$R^{2}$	RMSE
Onto2Vec	200	0.2019	189.54
OPA2Vec	200	0.3390	172.73
EL-embeddings	100	0.3008	177.64
Box2EL	200	0.3517	169.35
OWL2Vec*	200	0.2791	180.38
GOA2Vec	150	0.4201	160.77
Anc2Vec	200	0.2146	188.03
HiG2Vec	1000	0.5929	135.91
RotE	1000	0.6293	129.35
RefH	1000	0.6082	132.98
GeOKG-E	1000	**0.6487**	**125.92**

a MuRE and RefH are the best-performing KGE methods within the categories of Euclidean and hyperbolic embedding models, respectively.

#### 4.2.3 Multi-labeled interaction type classification

The STRING database categorizes interactions into seven types: activation, binding, catalysis, expression, inhibition, post-translational modification, and reaction. Since a protein pair can exhibit multiple interaction types, we formulate this as a multi-label classification task. Each interaction type is predicted with its own sigmoid output, and the model is optimized using a multi-label BCE loss. According to Table 7, GeOKG-E outperformed all other methods.

**Table 7.** STRING interaction type prediction. The best results are in bold.^a^

Model	Dim	Acc	Macro-F1	Micro-F1
Onto2Vec	200	0.3207	0.2845	0.6283
OPA2Vec	200	0.3866	0.3204	0.6909
EL-embeddings	100	0.3769	0.3126	0.6773
Box2EL	200	0.5339	0.3778	0.7777
OWL2Vec*	200	0.5044	0.3689	0.7610
GOA2Vec	150	0.4520	0.3337	0.7215
Anc2Vec	200	0.5532	0.3726	0.7940
HiG2Vec	1000	0.6926	0.4751	0.8639
RefE	1000	**0.7274**	**0.6074**	**0.8875**
RefH	1000	0.7168	0.5699	0.8822
GeOKG-E	1000	0.7133	0.5945	0.8794

a RotE and RotH are the best-performing KGE models within the categories of Euclidean and hyperbolic embedding models, respectively.

### 4.3 Generalization capability of gene embeddings on new data

We investigate whether the GO and gene embeddings, trained on the previous GOA data, can represent the newly added relationships. The GOA from June 2023 ($GOA_{2306}$) was compared with the GOA from April 2024 ($GOA_{2404}$), focusing on annotations supported by direct experimental evidence. GO terms with newly annotated genes were examined. Given a set of genes and GO terms within $GOA_{2306}$, $GO_{\mathrm{target}}$ is defined as the set of GO terms with new gene annotations in $GOA_{2404}$ but not in $GOA_{2306}$. For each term $t\in GO_{\mathrm{target}}$, we define:

-
New_genes : $\left\{u\;|\;u\in \mathrm{genes},\;(u,t)\in GOA_{2404}-GOA_{2306}\right\}$
-
Prev_genes : $\left\{u\;|\;(u,t)\in GOA_{2306}\right\}$
-
Other_genes : $\left\{u\;|\;u\in \mathrm{genes},(u,t)\notin GOA_{2404}\cup GOA_{2306}\right\}$

**Figure 3.**
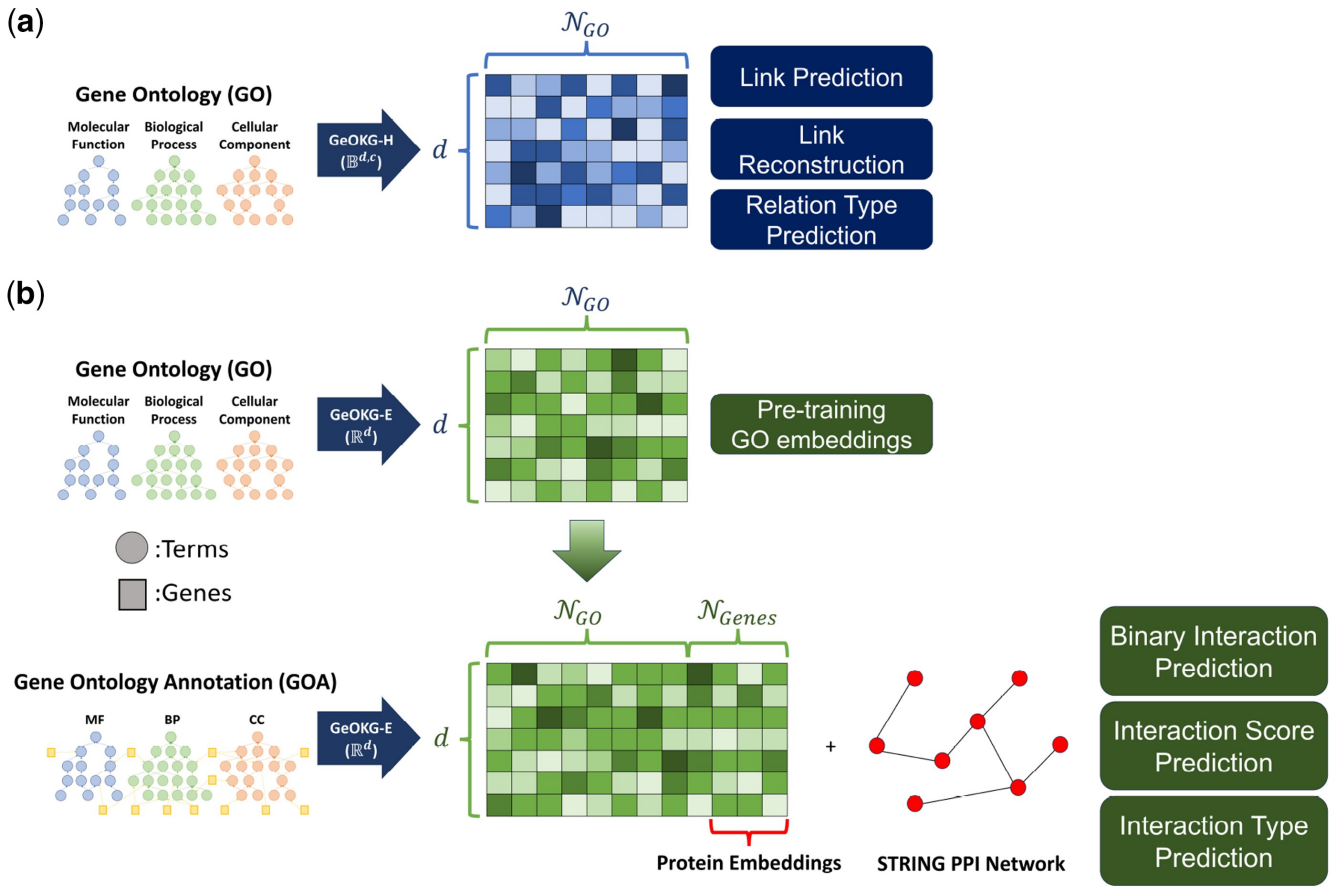
Overview of GeOKG. (a) GeOKG−H: Embeds the Gene Ontology (GO) graph in the hyperbolic space of a Poincaré ball ($B^{d,c}$) to preserve its hierarchical structure. Its effectiveness is validated through three GO-level tasks. (b) GeOKG−E: Targets the Gene Ontology Annotation (GOA) graph—comprising both GO terms and genes—using Euclidean space ($R^{d}$). This module uses a two-phase training process: initially pre-training on the GO graph to obtain baseline GO term embeddings, followed by fine-tuning on the GOA graph. Only genes corresponding to proteins in the STRING PPI network are selected, with the resulting protein embeddings evaluated via three protein–protein interaction prediction tasks. Here, $N_{GO}$ and $N_{\mathrm{Genes}}$ mean the number of GO terms and genes, respectively.

**Figure 4.**
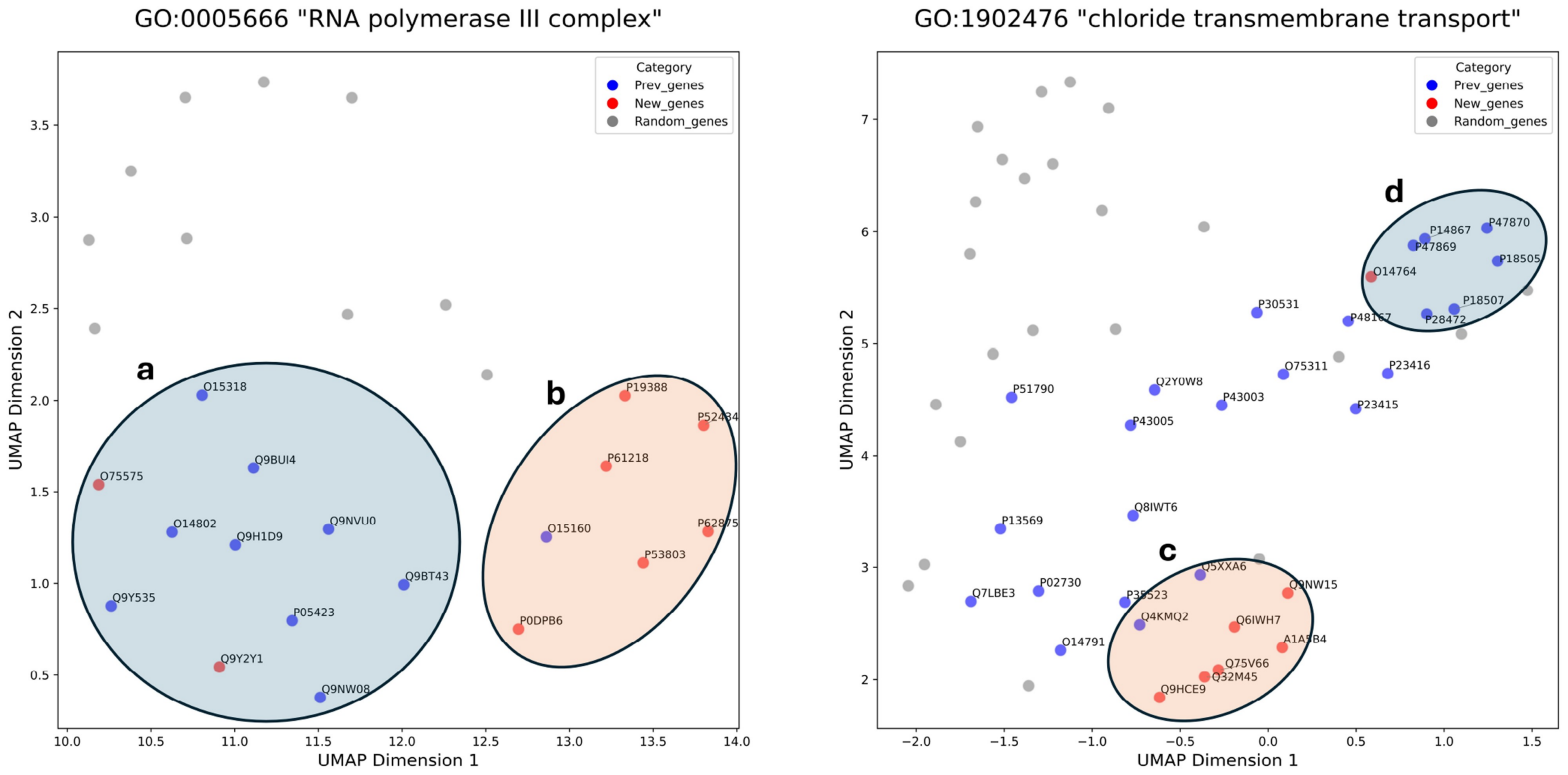
UMAP visualizations of gene embeddings. The UniProt ID of the gene product is labeled for New_genes and Prev_genes. Compared to Random_genes, Prev_genes are significantly closer to New_genes (*P*-values < 8.0e−30, Mann–Whitney test). We set “init” parameter as “random.”

Random_genes are randomly sampled from Other_genes in the same quantity as Prev_genes. The distances between New_genes and Prev_genes are significantly shorter than those between New_genes and Random_genes (Fig. 3, Supplementary Fig. S3). In Fig. 3, Prev_genes, New_genes, and Random_genes are represented by blue, red, and gray colors, respectively. All gene products in group **a** in Fig. 3 comprise components of RNA polymerase III, while those in group **b** consist of the common components of RNA polymerase I and III. Although they are part of the same cellular component, the RNA polymerase III complex, the embeddings of gene products exhibit a tendency to cluster based on shared features. Each member of the group **c** belongs to the Anoctamin family, whereas each gene product in group **d** is a subunit of the GABA recepter. Despite being annotated with the identical GO term and involved in the same biological process (chloride transmembrane transport), they also tend to cluster with gene products possessing similar features (Fig. 4).

## 5 Discussion

GeOKG extends the basic operations of TransE by integrating nonlinear transformations across multiple geometric spaces, which increases per-operation complexity. Despite these additional computations, the model maintains a comparable overall parameter count, making it more resource-efficient (Supplementary Section S5.3).

Our experiments confirm that the attention-based aggregation within GeOKG is crucial for optimally integrating information from various geometric spaces. Ablation studies reveal that replacing attention with simpler pooling methods degrades performance, and analysis of the learned attention weights shows a preferential focus on hyperbolic spaces—underscoring the advantage of the attention mechanism (Supplementary Section S5.4).

We conducted an ablation study to determine the optimal combination of interaction spaces. Notably, our experiments revealed that including the Euclidean space in the interaction model yields a significant performance increase. This finding aligns well with the insights gained from the Forman-Ricci curvature analysis of the GO graph (Supplementary Section S5.5).

The evolution of the learnable curvature parameters during training illustrates GeOKG’s ability to adapt its representation of the knowledge graph’s intrinsic geometry. One hyperbolic space converges to a low curvature value to effectively model strongly hierarchical structures, while the other trends toward a near-zero curvature to approximate nearly Euclidean regions, thereby validating the mixed geometric approach (Supplementary Section S5.6).

## 6 Conclusion

In this article, we introduced GeOKG, a method that leverages geometric interaction to learn vector representations capturing the complex, nonmonotonic hierarchy of the Gene Ontology (GO). By integrating information from multiple geometric spaces, GeOKG exhibits superior representational performance on GO compared to approaches confined to a single geometric framework. Moreover, its application to protein–protein interaction prediction demonstrated enhanced performance over several recent deep-learning methods. These results highlight the potential of geometric interaction for embedding heterogeneous biomedical ontologies.

## Supplementary Material

btaf160_Supplementary_Data

## Data Availability

The pre-trained representations of GO terms and genes with GeOKG-E model are available at https://doi.org/10.5281/zenodo.15165076.
