# Supplementary material for: GeOKG: geometry-aware knowledge graph embedding for Gene Ontology and genes
Source: Bioinformatics. 2025 Apr 11;41(4):btaf160. doi: 10.1093/bioinformatics/btaf160 (PMC12036960; doi:10.1093/bioinformatics/btaf160)
Supplement: btaf160_Supplementary_Data [file btaf160_supplementary_data.zip › [cleaned] GeOKG_supplementary.pdf]

Supplemetary materials for GeOKG

April 7, 2025

1 Forman-Ricci Curvature and Its Application to Gene Ontology Graphs

Forman-Ricci curvature is a discrete analogue of classical Ricci curvature, adapted for graphs and networks. Unlike the smooth manifold setting of classical Ricci curvature [Gu et al., 2018], Forman-Ricci curvature assigns a curvature value to each edge based on its weight and the connectivity of its endpoints. In unweighted networks, this measure simplifies to an expression such as

$$\kappa(e) = 2 - (\deg(v_1) + \deg(v_2)),$$

for an edge e connecting nodes v_1 and v_2 [Weber et al., 2017]. In this formulation, edges that connect high-degree nodes tend to have strongly negative curvature. Such negatively curved edges often indicate bottlenecks or bridges that play critical roles in maintaining the overall connectivity of the network, whereas edges within tightly knit clusters display near-zero or positive curvature, highlighting local cohesion.

In the context of Gene Ontology, where the graph represents hierarchical relationships among biological concepts, Forman-Ricci curvature offers a quantitative means to assess structural coherence and functional organization. Typically, edges linking broad parent GO terms to many specific child terms exhibit strongly negative curvature, reflecting the pronounced, tree-like branching structure of the hierarchy. In contrast, edges that connect closely related or functionally coherent terms tend to have higher curvature, suggesting denser local interconnections. This contrast enables us to discern distinct layers of organization within the GO graph. The diversity in curvature values observed in the GO graph (Figure S1) supports the assertion that the network possesses a complex structure.

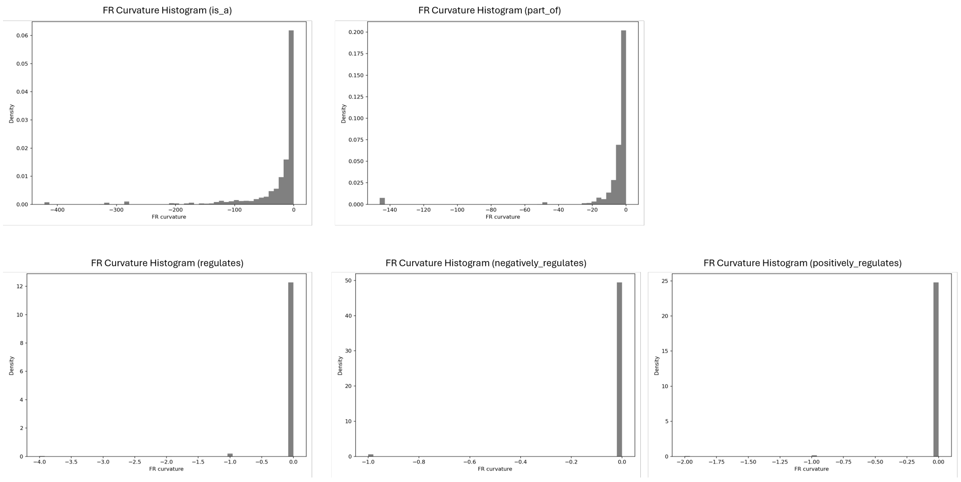

Figure S1: Distribution of Forman-Ricci Curvature from each relation graph of Gene Ontology.

2 Hyperbolic geometry

2.1 Exponential and logarithmic map

Suppose a hyperbolic space with a curvature $-c$ and dimension d, denoted as $\mathbb{B}^{d,c}$, for any point x within this space, $\mathbb{B}^{d,c}$ has a tangent space $T_x\mathbb{B}^{d,c}$. The exponential map $\exp_x^c : T_x\mathbb{B}^{d,c} \to \mathbb{B}^{d,c}$ and the logarithmic map $\log_x^c : \mathbb{B}^{d,c} \to T_x\mathbb{B}^{d,c}$ can be precisely described. Provided that v is not the zero vector and y is distinct from x, the mappings are represented as follows:

$$\exp_x^c(v) = x \oplus_c \left(\tanh\left(\sqrt{c}\frac{\lambda_x^c \|v\|}{2} \right) \frac{v}{\sqrt{c}\|v\|} \right), \tag{1}$$

$$\log_x^c(y) = \frac{2}{\sqrt{c}\lambda_x^c} \tanh^{-1}(\sqrt{c}\| - x \oplus_c y\|) \frac{-x \oplus_c y}{\| - x \oplus_c y\|}. \tag{2}$$

This lemma further expounds that these mappings assume more simplified forms when x equals the zero vector, for vectors v in the tangent space at the zero vector $T_{\mathbf{0}}\mathbb{B}^{d,c}$ not equal to zero, and for points y in $\mathbb{B}^{d,c}$ not equal to the zero vector:

$$\exp_{\mathbf{0}}^c(v) = \tanh(\sqrt{c}\|v\|)\frac{v}{\sqrt{c}\|v\|}, \tag{3}$$

$$\log_{\mathbf{0}}^c(y) = \tanh^{-1}(\sqrt{c}\|y\|)\frac{y}{\sqrt{c}\|y\|}. \tag{4}$$

Additionally, the exponential and logarithmic maps elucidate the transition to Euclidean geometry as the curvature $-c$ approaches zero. Specifically, the limit of $\exp_x^c(v)$ as c approaches zero is $x + v$, which aligns with the Euclidean exponential map, and similarly, the limit of $\log_x^c(y)$ as c converges to zero is $y - x$, congruent with the Euclidean

logarithmic map. This convergence underscores the geometric continuity between hyperbolic and Euclidean spaces.[Balazevic et al., 2019, Ganea et al., 2018]

2.2 Möbius operation

In the domain of $\mathbb{B}^{d,c}$, the operation known as Möbius addition, denoted as $x \oplus_c y$, is defined as follows:

$$x \oplus_c y := \frac{(1 + 2c\langle x, y\rangle + c|y|^2)x + (1 - c|x|^2)y}{1 + 2c\langle x, y\rangle + c^2|x|^2|y|^2}. \tag{5}$$

This operation extends the conventional Euclidean vector addition to a more complex geometric setting, reverting to the latter when the curvature parameter c approaches zero. It is noteworthy that for any positive value of c, the operation is neither commutative nor associative. Nonetheless, it adheres to specific axioms such as the identity and the left-cancellation law, alongside defining a corresponding Möbius subtraction operation. The Möbius subtraction is then defined by the use of the following notation: $\mathbf{x} \ominus_c \mathbf{y} = \mathbf{x} \oplus_c (-\mathbf{y})$.

Additionally, Möbius scalar multiplication for vectors in $\mathbb{B}^{d,c} \setminus \{\mathbf{0}\}$ with a scalar $r \in \mathbb{R}$ is introduced as:

$$r \otimes_c x := \frac{1}{\sqrt{c}} \tanh\left(r \tanh^{-1}(\sqrt{c}|x|)\right) \frac{x}{|x|}, \tag{6}$$

providing a scalable operation that converges to traditional scalar multiplication in Euclidean space as c diminishes to zero. This operation exhibits properties such as distributivity and associativity with respect to scalar multiplication, as well as a scaling property concerning the norm of vectors.

3 Relational operations in Knowledge graph embeddings

Knowledge graph embedding (KGE) models incorporate relational information by applying different vector operations to entity embeddings. In Euclidean space, for instance, TransE models a relation as a translation, where the relation vector is added to the head entity vector, i.e., $e_t = e_h + e_r$. In contrast, MuRE employs Hadamard (element-wise) multiplication between the head entity vector and the relation vector, resulting in $e_t = e_h \odot e_r$.

When extending these operations to hyperbolic space, it is essential that the operations respect the underlying geometry. For example, TransP is the hyperbolic analogue of TransE. Instead of standard vector addition, TransP uses Möbius addition—a hyperbolic equivalent of addition. In this approach, the head entity vector is first mapped into hyperbolic space via the exponential map and then combined with the relation vector us-

ing Möbius addition. Similarly, MuRP adapts the MuRE model by replacing Euclidean Hadamard multiplication with Möbius scalar multiplication; here, the relation vector interacts with the mapped head entity vector in a manner that is consistent with hyperbolic geometry.

In addition to these models, approaches such as RotE and RefE utilize geometric isometries—specifically, rotations and reflections—to encode relational properties like symmetry and anti-symmetry. Their hyperbolic counterparts, RotH and RefH, extend these operations to hyperbolic space while preserving the intrinsic distance metrics.

These rotations and reflections are implemented as relation-specific isometries. Let the relation-specific parameters be defined as $\Theta_r = (\theta_{r,i})_{i=1}^{d/2}$ and $\Phi_r = (\phi_{r,i})_{i=1}^{d/2}$ for an even-dimensional embedding space with dimension d. The rotation and reflection operations are parameterized by block-diagonal matrices:

$$Rot(\Theta_r) = \text{diag}(G^{+}(\theta_{r,1}), ..., G^{+}(\theta_{r,d/2})), \tag{7}$$

$$Ref(\Phi_r) = \text{diag}(G^{-}(\phi_{r,1}), ..., G^{-}(\phi_{r,d/2})), \tag{8}$$

where the 2×2 matrices $G^{\pm}(\theta) = \begin{bmatrix} cos(\theta) & \mp sin(\theta) \\ sin(\theta) & \pm cos(\theta) \end{bmatrix}$.

These matrices are hyperbolic isometries, which means they can be directly applied to hyperbolic embeddings without distorting the geometry.

4 Materials

4.1 Processing Gene Ontology (GO)

Gene Ontology can be downloaded from 'http://geneontology.org/docs/download-ontology/'. For our study, we used go-basic.obo (version 1.2, released/2023-06-11). This version of GO contains the core GO, filtered such that the graph is guaranteed to be acyclic and annotations can be propagated up the graph. The relationships between Gene Ontology (GO) terms were extracted from the GO dataset and transformed into triplets of the form (h, r, t), for instance, (*nucleotide binding*, *is_a*, *nucleoside phosphate binding*). During this phase, a Gene Ontology (GO) term was excluded if it was marked as *is_obsolete*. The resulting Gene Ontology (GO) corpus comprised a total of 83,975 edges across 42,950 GO terms. The composition of edges for each relation type is as follows — *is_a*: 68650, *part_of*: 6809, *regulates*: 3120, *negatively_regulates*: 2704, and *positively_regulates*: 2692. For the dataset construction aimed at the GO level task experiment, the data were initially split into training, validation, and test sets in a ratio of 8:1:1. To ensure balanced training for the relation type prediction task, the splitting was performed in a stratified manner based on the type of relation. In addition, for the link reconstruction experiment,

the method of hard negative sampling was employed during the process of negative sampling. For a positive pair (h,t), a node that is within a 3-hop distance from h in the GO graph but does not have a direct relationship with h is randomly sampled to perturb the t, thereby forming the negative pair. In this strategy, negative pairs are sampled in a quantity equivalent to that of the positive pairs. The rationale behind employing this hard negative sampling technique stems from the observation that Knowledge Graph Embedding (KGE) methods demonstrated exceedingly high performance in link reconstruction tasks, rendering negligible differences in performance across models. To discern performance differentials between models, experiments were designed under more challenging scenarios.

4.2 Processing Gene Ontology Annotation (GOA)

Similarly to the approach taken with the GO dataset, triplets of the form (h,r,t) were extracted from the GOA dataset, for example, (*moeA5*, *involved_in*, *biosynthetic process*). Within the annotations present in the GOA, we excluded those annotated with the evidence code ND, denoting the absence of biological data. The resulting GOA corpus comprised a total of 286,628 interactions across 18,137 genes and 42,950 GO terms. The splitting process was conducted in a manner similar to that of the GO dataset, employing the same ratio and stratified approach.

4.3 Processing STRING Protein-protein interaction network

STRING (Search Tool for the Retrieval of Interacting Genes/Proteins) is a comprehensive database that catalogs both known and predicted protein-protein interactions. It has aggregated interaction data across over 2,000 species, deriving from five principal sources: genomic context predictions, high-throughput lab experiments, co-expression, automated text mining, and previous knowledge in databases. Our research specifically focused on the protein-protein interactions (PPIs) pertinent to Homo sapiens.

For the binary interaction classifying task, we utilized STRING PPI full network (version 11.5, released August 12, 2021). The interaction is assigned a confidence score, denoting the estimated likelihood of the existence of a predicted association, with values spanning from 0 to 1,000. The links with a confidence score of 900 or higher are filtered from the PPI network. These filtered links are designated as positive links, while the remaining links are considered negative links. An equivalent number of negative links are then randomly sampled to match the quantity of positive links.

In the binding affinity prediction task, we utilized PPI physical subnetwork (version 11.5, released August 12, 2021). Within the physical network, physical interaction scores are attributed to associations in protein-protein interactions (PPI) if there is evidence indicating the concurrent presence of the proteins within a complex.

For interaction type classification, we utilized PPI subnetwork with interaction types for protein links (version 11.0, released January 19, 2019). STRING no longer offers the actions mode due to a variety of reasons, primarily because its maintenance and support demanded substantial resources, thereby impeding the advancement of other segments of STRING. Consequently, compared to preceding tasks, previous versions of the data were utilized, and the latest data offering the action mode was employed.

5 Methods

5.1 GeOKG-E

GeOKG-E is the model that embeds the Gene Ontology Annotation (GOA) graph, which consists of GO terms and genes, accounts for the non-hierarchical nature of relationships between genes and GO terms. Consequently, this model is embedded in Euclidean space and is referred to as GeOKG-E. (Figure S2)

The methodology of GeOKG-E can be summarized into four main steps:

1. Initialize the head and tail embeddings (e_h, e_t) in Euclidean space.
2. Use exponential maps to project the head entity from Euclidean space $\mathbb{R}^d$ to hyperbolic interaction spaces $\mathbb{B}^{d,c_1}$ and $\mathbb{B}^{d,c_2}$.
3. In each space, apply rotational transformation $Rot(\cdot)$ and aggregate the results back into Euclidean space.
4. Utilize an attention mechanism, $att(\cdot)$ to fuse the geometric information from both Euclidean and hyperbolic spaces. And then project the result and tail entity (e_t) into the hyperbolic space with curvature $-c$ (H_h^c)
5. In the hyperbolic space with curvature $-c$, apply the translational operation with e_r to H_h^c so that it moves closer to the projected tail entity H_t^c.

For a head entity h, the embedding transformations are given by:

- $E_h = Rot(\Theta_r)e_h$ for the Euclidean space embedding,
- $H_h^{c_1} = Rot(\Theta_r)\exp_0^{c_1}(e_h)$ for the first hyperbolic space embedding, where $\exp_0^{c_1}(e_h)$ (with $c_1 > 0$) obtains the projection of the head entity into the first hyperbolic interaction space,
- $H_h^{c_2} = Rot(\Theta_r)\exp_0^{c_2}(e_h)$ for the second hyperbolic space embedding, where $\exp_0^{c_2}(e_h)$ (with $c_2 > 0$) obtains the projection of the head entity into the second hyperbolic interaction space.

where $Rot(\cdot)$ and Θ_r are the rotational function and parameter, respectively (Section 3).

The subsequent scoring and loss computation procedures follow the same approach as in GeOKG-H.

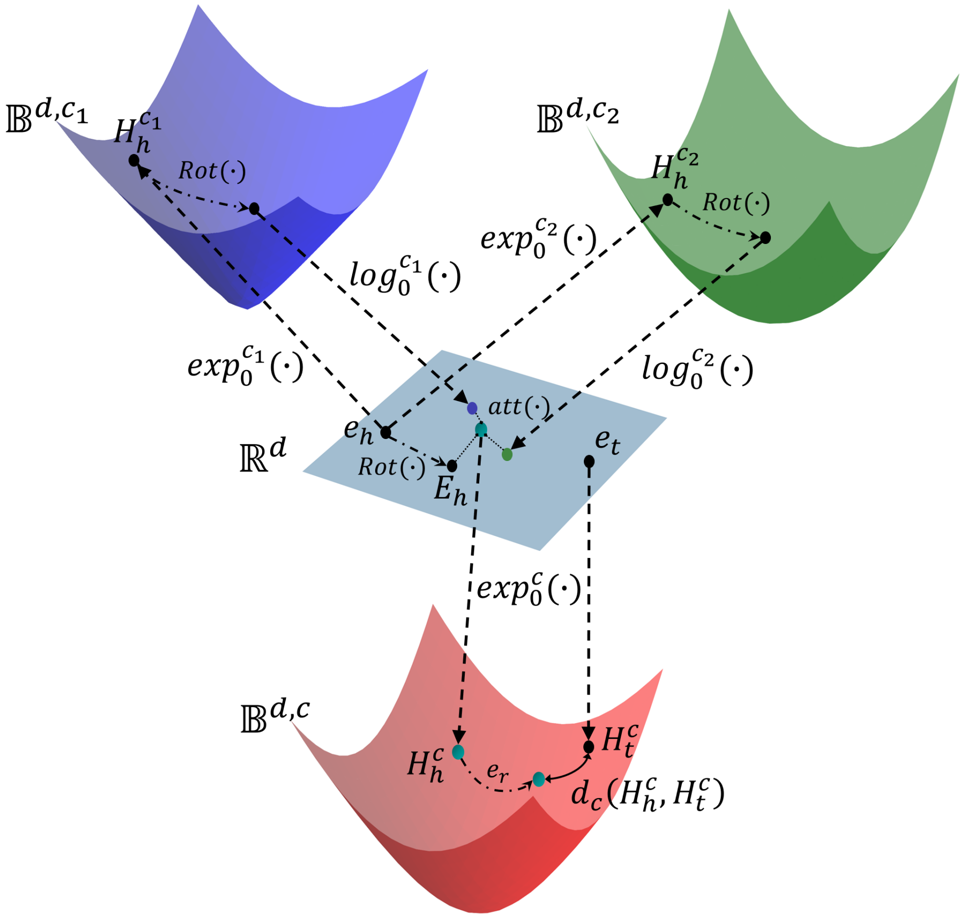

Figure S2: Schematic of GeOKG-E. GeOKG-E is a Euclidean space embedding model for GOA.

e_h and e_t are the final embeddings of head and tail entities, respectively. The approach uses exponential and logarithmic mappings to bridge between Euclidean and hyperbolic spaces. In each interaction space, the rotational transformation $Rot(\cdot)$ is applied and then the results are integrated back into the Euclidean space. The attention mechanism, $att(\cdot)$, aggregates the geometric information from all the interaction spaces. The learning objective in hyperbolic space with learnable curvature c is to optimize the entity and relation embeddings by minimizing the hyperbolic distance $d_c(H_h^c, H_t^c)$. Throughout the optimization process, the head and tail embeddings in Euclidean space are updated.

5.2 Embedding dimension and hyperparameter

We conducted an extensive grid search to determine all key hyperparameters, including embedding dimension, negative sampling size, loss regularizer, and learning rate. For the

negative sampling size, we evaluated values of 1, 10, 50, and 100 negatives per positive sample. Similarly, the loss regularizer was varied among 0, 0.01, and 0.1, and the learning rate among 0.0001, 0.001, and 0.01. Our grid search identified the optimal setting as a negative sampling size of 50, a loss regularizer of 0, and a learning rate of 0.001. The embedding dimension and hyperparameters for each model is detailed in Table S1

Table S1: Embedding dimension and hyperparameter.

model	Dim	Negative sample size	Loss regularizer	Learning rate
GeOKG-H	300	50	0	0.001
GeOKG-E	1000	50	0	0.001

5.3 Computational complexity of GeOKG compared to other methods

Compared to the basic Knowledge Graph Embedding (KGE) model TransE, GeOKG involves more complex computational operations. Whereas TransE primarily relies on simple vector addition and Euclidean distance calculations, GeOKG performs intricate vector transformations across multiple spaces, applies attention-weighted summation, and computes distances through nonlinear operations. Specifically, the use of exponential and logarithmic maps for space transformations, in conjunction with the *tanh* function for distance evaluations, introduces significant nonlinearity that increases computational time.

In terms of learnable parameters, GeOKG only introduces a modest increase relative to TransE. TransE's parameters consist solely of entity embeddings ($|V| \times d$) and relation embeddings ($|R| \times d$), totaling $((|V| + |R|) \times d)$, where V is the set of entities and R is the set of relation types. In contrast, GeOKG adds curvature parameters ($3|R|$), rotation parameters ($|R| \times d$), and attention parameters ($|R| \times d$), resulting in a total parameter count of $((|V| + 2|R|) \times d) + (|R| \times 3)$. Given that $|V|$ is typically much larger than $|R|$, the overall increase in parameters is relatively small. For Gene Ontology, $|V| = 49,250$ and $|R| = 5$.

Performance considerations further underscore GeOKG's efficiency. For example, in Gene Ontology (GO)-level link prediction experiments, the RotE model required an embedding dimension of 1,000 to achieve performance lower than that of GeOKG, which only used a 200-dimensional embedding (Table S2). This suggests that alternative models may demand substantially more computational resources to reach comparable performance levels.

Table S2: Link prediction performance across various dimensions. The evaluation metric is MRR.

Dim	GeOKG-H	RotH	RotE
10	0.246	0.225	0.159
20	0.329	0.326	0.296
50	0.367	0.369	0.359
100	0.380	0.376	0.373
200	0.383	0.381	0.378
300	**0.385**	0.378	0.381
500	0.383	0.378	0.376
1000	0.381	0.376	0.382

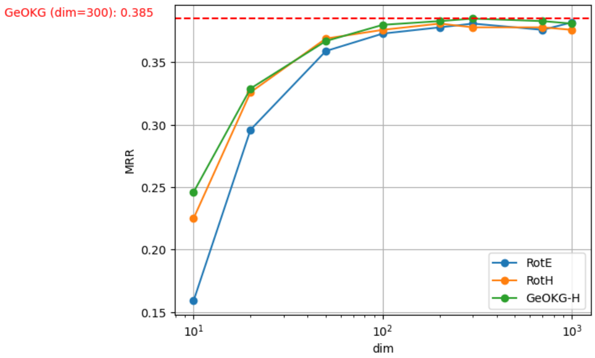

Figure S3: Impact of embedding dimension.

5.4 Impact of attention mechanism

We investigated the impact of the attention mechanism in the geometric interaction on model performance. We evaluated its contribution through an ablation study by replacing the attention-based aggregation (Att) with alternative strategies—specifically, max pooling (Max) and average pooling (Avg)—across the different interaction spaces with 300-dimension. Our experiments consistently demonstrated that the attention mechanism delivers superior performance compared to these alternatives. (Table S3).

Furthermore, we examined the learned attention weights across various relation types. The analysis revealed that the model allocates more attention to hyperbolic spaces than to the Euclidean space, reflecting the underlying hierarchical structure of the GO graph (Figure S4).

Table S3: Ablation study on attention mechanism.

Aggregation	MRR	H@1	H@10	H@50
Max	0.376	0.277	0.573	0.698
Avg	0.380	0.280	0.578	0.702
Att	**0.385**	**0.288**	**0.581**	**0.704**

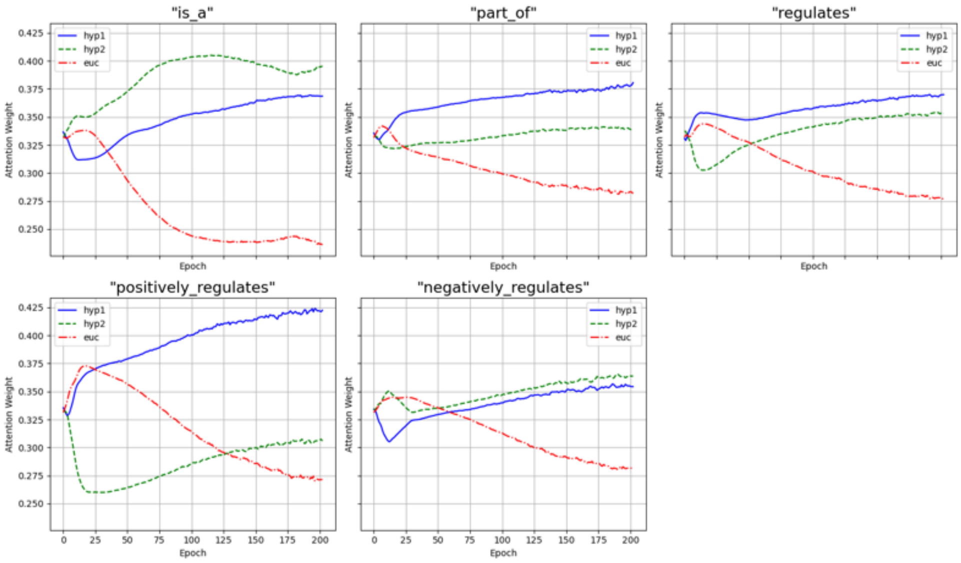

Figure S4: Attention weights across training epoch for each relation type. "hyp1" and "hyp2" mean two different hyperbolic interaction spaces. "euc" denotes a Euclidean interaction spaces.

5.5 Study on the combination of the interaction spaces

Previous studies and experimental results have confirmed the advantages of geometry interaction [Cao et al., 2022, Zhu et al., 2020]. However, the optimal configuration of the interaction space remains an open question. We designed our model by heuristically testing various combinations of interaction spaces and selecting the best combination. As shown in Table S4, the results indicate that the combination of one Euclidean space and two hyperbolic spaces yielded the best outcomes.

Our ablation studies on the geometric interaction module reveal that incorporating the Euclidean space significantly enhances overall performance. Specifically, when comparing models that use only a single geometric space with our mixed-space approach, we found that combining Euclidean and hyperbolic geometries leads to markedly improved link prediction performance. This result indicates that the model capitalizes on the complementary strengths of each geometry: the Euclidean space offers stability and simplicity in regions with minimal curvature, while the hyperbolic space effectively captures

the hierarchy. This finding aligns well with the insights gained from the Forman-Ricci curvature analysis that the GO graph has complex hierarchical patterns (Figure S1).

Table S4: Ablation study on the combination of the interaction spaces with 300-dimension in GeoKG-H

Interaction space	MRR	H@1	H@10	H@50
$\mathbb{E}$	0.381	0.283	0.579	0.702
$\mathbb{H}$	0.378	0.280	0.570	0.700
$\mathbb{HH}$	0.380	0.280	<u>0.581</u>	0.705
$\mathbb{HHH}$	0.376	0.276	0.577	0.700
$\mathbb{EH}$	<u>0.383</u>	<u>0.285</u>	0.577	<u>0.707</u>
$\mathbb{EHH}$	**0.385**	**0.288**	<u>0.581</u>	0.704
$\mathbb{EHHH}$	0.378	0.275	**0.582**	**0.708**

5.6 Learning curvature parameter

In Figure S5, we present the evolution of the learnable curvature parameters for the GO graph over the training epochs. Our findings reveal that, as the loss is minimized, the model distinctly differentiates between two hyperbolic interaction spaces. One space is driven toward a markedly low curvature value, capturing strongly hierarchical structures, while the other converges to a curvature value near zero, effectively approximating Euclidean geometry. The divergence in the learned parameters closely mirrors the wide dispersion observed in the Forman–Ricci curvature estimates for the GO graph (Figure S1). This behavior demonstrates that the model dynamically allocates one hyperbolic space to encode pronounced hierarchical relationships and another to model regions with nearly Euclidean characteristics. Such differentiation underscores the benefits of employing a mixed geometric approach, as it allows the embedding process to adapt to the graph's inherently heterogeneous structural properties.

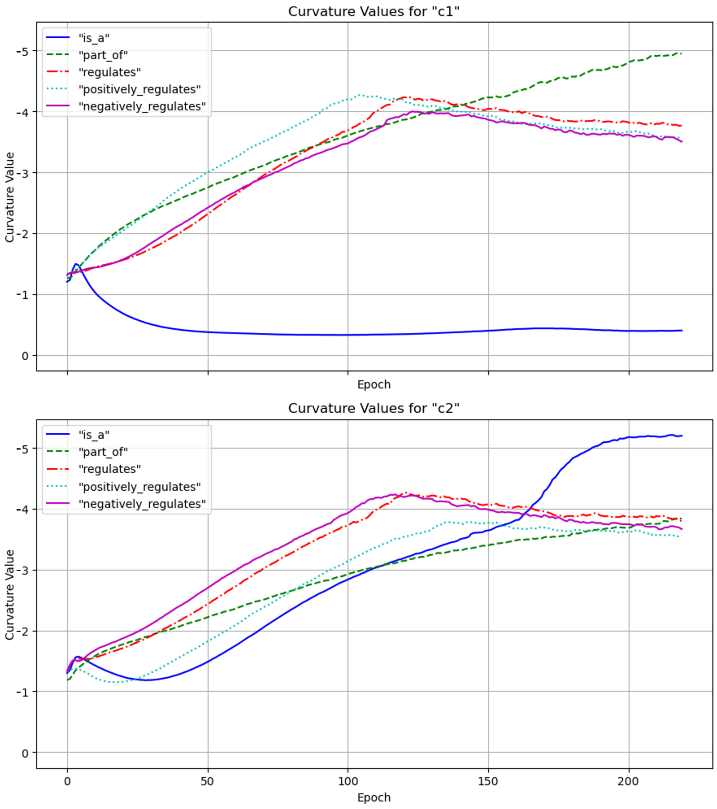

Figure S5: Learnable curvature values across training epochs from hyperbolic interaction spaces

6 Results

6.1 Broader comparison results from GO-level experiments

GeOKG achieves superior performance in both link prediction (Table S5) and link reconstruction (Table S6). While AttH excels in relation type prediction (Table S7), the primary strength of GeOKG lies in its robust generalization across a variety of tasks. Furthermore, when comparing models that utilize the same relational operation, RotE and RotH, GeOKG outperforms the other models.

$\mathbb{R}^d$ represents the Euclidean space, and $\mathbb{B}^d$ represents the Poincaré ball. $\mathcal{U}$ is the utilized space for vector embedding or geometry interaction. The best results are in bold, and the second-best results are underlined.

Table S5: Link prediction results on Gene Ontology.

$\mathcal{U}$	Model	Dim	MRR	H@1	H@10	H@50
$\mathbb{R}^d$	TransE	300	0.266	0.164	0.474	0.641
$\mathbb{R}^d$	MuRE	500	0.360	0.262	0.558	0.693
$\mathbb{R}^d$	RefE	500	0.338	0.238	0.540	0.688
$\mathbb{R}^d$	RotE	1000	<u>0.382</u>	0.283	<u>0.577</u>	**0.706**
$\mathbb{R}^d$	AttE	300	0.380	0.279	0.576	0.700
$\mathbb{B}^d$	TransP	500	0.273	0.171	0.486	0.663
$\mathbb{B}^d$	MuRP	300	0.339	0.241	0.535	0.682
$\mathbb{B}^d$	RefH	300	0.348	0.250	0.542	0.686
$\mathbb{B}^d$	RotH	200	0.381	0.283	0.574	0.694
$\mathbb{B}^d$	AttH	200	0.376	0.283	0.560	0.686
$\mathbb{R}^d, \mathbb{B}^d$	**GeOKG-H**	300	**0.385**	**0.288**	**0.581**	<u>0.704</u>

Table S6: Link reconstruction results on Gene Ontology.

$\mathcal{U}$	Model	Dim	AUROC	AUPRC	F1 score
$\mathbb{R}^d$	TransE	200	0.908	0.932	0.837
$\mathbb{R}^d$	MuRE	300	0.928	0.946	0.861
$\mathbb{R}^d$	RefE	200	0.898	0.927	0.839
$\mathbb{R}^d$	RotE	300	0.901	0.929	0.835
$\mathbb{R}^d$	AttE	300	0.925	0.943	0.851
$\mathbb{B}^d$	TransP	100	0.909	0.933	0.839
$\mathbb{B}^d$	MuRP	500	0.917	0.939	0.852
$\mathbb{B}^d$	RefH	100	0.895	0.926	0.839
$\mathbb{B}^d$	RotH	300	0.905	0.931	0.842
$\mathbb{B}^d$	AttH	100	<u>0.928</u>	<u>0.946</u>	<u>0.863</u>
$\mathbb{R}^d, \mathbb{B}^d$	**GeOKG-H**	1000	**0.945**	**0.956**	**0.868**

Table S7: Relation type prediction results on Gene Ontology.

$\mathcal{U}$	Model	Dim	Macro F1	Micro F1
$\mathbb{R}^d$	TransE	100	0.796	0.861
$\mathbb{R}^d$	MuRE	500	<u>0.867</u>	<u>0.932</u>
$\mathbb{R}^d$	RefE	300	0.823	0.918
$\mathbb{R}^d$	RotE	1000	0.846	0.920
$\mathbb{R}^d$	AttE	1000	0.771	0.783
$\mathbb{B}^d$	TransP	100	0.797	0.842
$\mathbb{B}^d$	MuRP	100	0.846	0.917
$\mathbb{B}^d$	RefH	300	0.839	0.903
$\mathbb{B}^d$	RotH	300	0.851	0.906
$\mathbb{B}^d$	AttH	300	**0.942**	**0.963**
$\mathbb{R}^d, \mathbb{B}^d$	**GeOKG-H**	100	0.865	0.924

6.2 Broader comparison from PPI prediction experiments

GeOKG-E consistently outperformed the competing methods on both the PPI binary classification (Table S8) and score prediction tasks(Table S9). However, GeOKG-E exhibited relatively lower performance in the type prediction task (Table S10). We attribute this outcome to the GeOKG training objective, which is specifically designed to cluster proteins with related functions in the embedding space. While this design is beneficial for capturing functional similarities, it does not directly address the challenges associated with accurately predicting protein-protein interaction types. Despite this limitation, GeOKG-E still markedly outperforms the conventional GO embedding method and demonstrates superior performance on the remaining PPI prediction tasks.

Table S8: STRING binary interaction prediction

**Model**	**Dim**	**AUROC**	**AUPRC**	**F1 score**
TransE	1000	0.9831	0.9829	0.9405
MuRE	1000	0.9821	0.9822	0.9383
RefE	1000	0.9836	0.9837	0.9414
RotE	1000	0.9833	0.9833	0.9408
AttE	1000	0.9832	0.9828	0.9406
TransP	1000	0.9821	0.9819	0.9382
MuRP	1000	0.9810	0.9808	0.9373
RefH	1000	0.9825	0.9822	0.9394
RotH	1000	0.9830	0.9828	0.9398
AttH	1000	0.9821	0.9819	0.9381
Onto2Vec	200	0.9314	0.9313	0.8586
OPA2Vec	200	0.9629	0.9621	0.9051
EL-embeddings	100	0.9339	0.9336	0.8614
Box2EL	200	0.9724	0.9727	0.9189
OWL2Vec*	200	0.9633	0.9624	0.9033
GOA2Vec	150	0.9401	0.9402	0.8718
Anc2Vec	200	0.9712	0.9716	0.9194
HiG2Vec	1000	0.9787	0.9790	0.9301
GeOKG-E	1000	**0.9848**	**0.9849**	**0.9455**

Table S9: STRING physical interaction score prediction

**Model**	**Dim**	R^2	**RMSE**
TransE	1000	0.6267	129.80
MuRE	1000	0.6202	130.93
RefE	1000	0.6327	128.75
RotE	1000	0.6293	129.35
AttE	1000	0.6288	129.44
TransP	1000	0.6076	133.08
MuRP	1000	0.5836	137.10
RefH	1000	0.6082	132.98
RotH	1000	0.6049	133.54
AttH	1000	0.6061	133.33
Onto2Vec	200	0.2019	189.54
OPA2Vec	200	0.3390	172.73
EL-embeddings	100	0.3008	177.64
Box2EL	200	0.3517	169.35
OWL2Vec*	200	0.2791	180.38
GOA2Vec	150	0.4201	160.77
Anc2Vec	200	0.2146	188.03
HiG2Vec	1000	0.5929	135.91
GeOKG-E	1000	**0.6487**	**125.92**

Table S10: STRING interaction type prediction

**Model**	**Dim**	**Acc**	**Macro-F1**	**Micro-F1**
TransE	1000	0.7067	0.5952	0.8759
MuRE	1000	0.7197	0.5682	0.8842
RotE	1000	0.7217	0.5726	0.8853
RefE	1000	**0.7274**	**0.6074**	**0.8875**
AttE	1000	0.7137	0.5765	0.8799
TransP	1000	0.7024	0.5572	0.8749
MuRP	1000	0.6951	0.4794	0.8664
RotH	1000	0.7146	0.5685	0.8818
RefH	1000	0.7168	0.5699	0.8822
AttH	1000	0.7131	0.5681	0.8810
Onto2Vec	200	0.3207	0.2845	0.6283
OPA2Vec	200	0.3866	0.3204	0.6909
EL-embeddings	100	0.3769	0.3126	0.6773
Box2EL	200	0.5339	0.3778	0.7777
OWL2Vec*	200	0.5044	0.3689	0.7610
GOA2Vec	150	0.4520	0.3337	0.7215
Anc2Vec	200	0.5532	0.3726	0.7940
HiG2Vec	1000	0.6926	0.4751	0.8639
GeOKG-E	1000	0.7133	0.5945	0.8794

6.3 Generalization Capability of Gene Embeddings on New Data

We selected the top 100 GO terms with the highest number of **New_genes** from GO_{target}. For each $t \in GO_{target}$, the distance between the groups of **New_genes** and **Prev_genes** is then compared to the distance between the groups of **New_genes** and **Random_genes**. The distance between two groups is calculated as the average distance between points in one group and points in the other group.

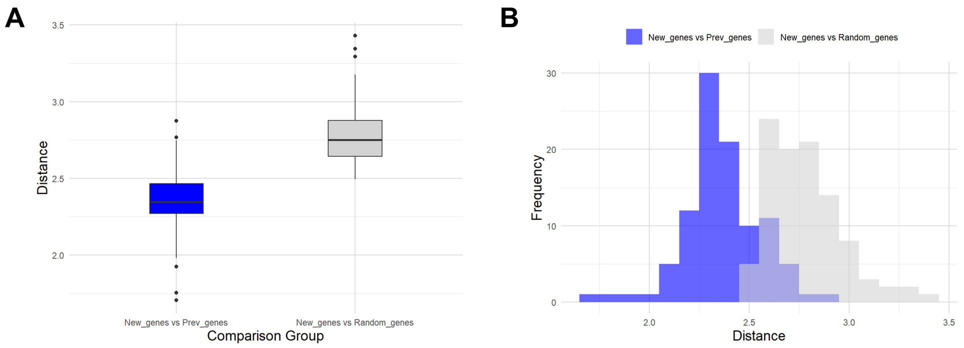

Figure S6: Comparison of Distances Between Two Groups

The distances between **New_genes** and **Prev_genes** are expected to be shorter than those between **New_genes** and **Random_genes**. Compared to **Random_genes**, **Prev_genes** demonstrated significantly greater proximity to New genes (Figure S6, p-values ¡ 8.0e-30, Mann-Whitney test).

References

Ivana Balazevic, Carl Allen, and Timothy Hospedales. Multi-relational poincaré graph embeddings. *Advances in Neural Information Processing Systems*, 32, 2019.

Zongsheng Cao, Qianqian Xu, Zhiyong Yang, Xiaochun Cao, and Qingming Huang. Geometry interaction knowledge graph embeddings. In *Proceedings of the AAAI Conference on Artificial Intelligence*, volume 36, pages 5521–5529, 2022.

Octavian Ganea, Gary Bécigneul, and Thomas Hofmann. Hyperbolic neural networks. *Advances in neural information processing systems*, 31, 2018.

Albert Gu, Frederic Sala, Beliz Gunel, and Christopher Ré. Learning mixed-curvature representations in product spaces. In *International conference on learning representations*, 2018.

Melanie Weber, Emil Saucan, and Jürgen Jost. Characterizing complex networks with forman-ricci curvature and associated geometric flows. *Journal of Complex Networks*, 5:527–550, 8 2017. ISSN 20511329. doi: 10.1093/comnet/cnw030.

Shichao Zhu, Shirui Pan, Chuan Zhou, Jia Wu, Yanan Cao, and Bin Wang. Graph geometry interaction learning. *Advances in Neural Information Processing Systems*, 33:7548–7558, 2020.
